# Sensory Properties of Fermented Blends of Sunflower Press Cake and Whey

**DOI:** 10.3390/foods14091489

**Published:** 2025-04-24

**Authors:** Harald Rohm, Sophie Morejón Caraballo, Ana Salvador, Sofia Mendo, Empar Llorca, Stefano Cattaneo, Ivano De Noni, Susanne Struck, Roberto Foschino, Isabel Hernando

**Affiliations:** 1 Chair of Food Engineering, Institute of Natural Materials Technology, Technische Universität Dresden, 1069 Dresden, Germany; sophie.morejon_caraballo@tu-dresden.de (S.M.C.); susanne.struck@th-owl.de (S.S.); 2 Institute of Agrochemistry and Food Technology (IATA-CSIC), 46980 Valencia, Spain; asalvador@iata.csic.es; 3 Department of Biomedical, Surgical and Dental Sciences, Università degli Studi di Milano, 20133 Milano, Italy; sofia.mendo@unimi.it (S.M.); roberto.foschino@unimi.it (R.F.); 4 Instituto Universitario de Ingeniería de Alimentos—Food UPV, Universitat Politècnica de València, 46022 Valencia, Spain; emllomar@tal.upv.es (E.L.); mihernan@tal.upv.es (I.H.); 5 Department of Food, Environmental and Nutritional Sciences, Università degli Studi di Milano, 20129 Milan, Italy; stefano.cattaneo@unimi.it (S.C.); ivano.denoni@unimi.it (I.D.N.)

**Keywords:** food losses, sustainability, by-product utilization, spreadability

## Abstract

Sustainability in the food chain and the prevention of food losses is an issue of increasing importance. There is a large number of processing by-products where innovative strategies are helpful for transferring these losses into a consumable state. In a step-by-step approach, this current study focused on the sensory properties of blends of sunflower press cake and whey, fermented with different consortia of microorganisms and intended for being used as a basis for a savory spread. In the first part of the work, blends fermented with six co-cultures from lactic acid bacteria and yeasts were characterized by free choice profiling and quantitative descriptive analysis. The respective results were used to modify the formulation and to select the fermentation cultures that were promising from a sensory point of view. Subsequent investigations allowed reducing sample dimensionality further, and the study was concluded by affective hedonic tests and a check-all-that-apply set-up performed by consumers. The final experiment also comprised a just-about-right approach performed for specifically evaluating spreadability. The outcome of the entire study indicates that it is possible to tailor attractive foods from by-products, provided that the need for final optimizations regarding palatability is considered.

## 1. Introduction

In the last decade, sustainability has become a major issue also for the food industry, and numerous research projects have been carried out in this field. An impressive example of a platform responsible for implementing strategic research and development is SUSFOOD and its successor (https://susfood-db-era.net, accessed on 25 February 2025). In the five calls up to now, the main research topics covered all aspects of resource efficiency, added value given to by-products, the reduction of production losses and food waste, new sustainable technologies as well as new raw materials for foods and organic food production and consumer-related aspects (e.g., [1,2,3,4,5,6,7]). One of the projects that was funded in conjunction with the CORE Organic Cofund (https://projects.au.dk/coreorganiccofund/ accessed on 25 February 2025) and in the framework of the ‘Towards sustainable and organic food systems’ call in 2019 was FERBLEND, with the aim ‘… to explore the potential of creating synergies between two side streams, sunflower press cake and cheese whey, and their upcycling using fermentation combined with other processes.’ [8].

The leftover from sunflower oil production is still underexploited for human consumption and, in line with many other by-products, currently mainly intended to be used as animal feed or in the production of fertilizers, energy or other materials [9]. This press cake represents a valuable source of dietary fiber (approx. 40 g/100 g) and protein, up to 48 g/100 g when oil production involves both pressing and solvent extraction [10]. When only mechanical pressing is applied, the residual oil content in the press cake may be up to 24 g/100 g [11], and protein and fiber content is proportionally lower. For press cake from sunflower and other oil seeds, protein extraction for further use was addressed in a large number of studies (e.g., [12,13,14,15]). However, reports on the direct use of press cake as a food constituent are scarce [16,17].

In the European Union, the amount of whey as a by-product from rennet or acid-curd cheese production is approx. 55 million tons [18]. Half of this amount is estimated to be transformed by large dairy companies either directly into whey powder or to serve as a substrate for obtaining whey ingredients, mainly protein and lactose, for being further used in food or pharmaceutical formulations [19,20,21]. However, artisanal, small- and medium-sized cheese manufacturers do not have the equipment for the necessary processes so that it is used as feed, for biogas conversion or simply discarded [22,23].

In the FERBLEND project, research groups from six countries placed emphasis on the pre-treatment of the raw materials and blends thereof, on fermentation aspects, and on the use of fermented blends (see, for example, [24,25,26,27,28,29,30]). The aim of this current study was to evaluate the sensory properties of press cake/whey blends fermented by different microorganisms in a step-by-step design to assist in finding a sound solution for its use as a food ingredient.

## 2. Materials and General Methods

### 2.1. Materials

Sunflower seed press cake (PC), the remnant of oil production from hulled sunflower seeds by mechanical pressing, was obtained from Schalk Mühle GmbH & Co. KG (Ilz, Austria). The PC, provided in milled state, contained 8.34 g/100 g moisture and, per 100 g dry matter, 43.14 g protein, 9.85 g residual fat, 7.89 g ash and 19.45 g dietary fiber [24]. Bovine sweet whey powder with 12.1 g/100 g protein, 0.9 g/100 g fat, 73.1 g/100 g total carbohydrates and 7.9 g/100 g ash [25] was from Bayerische Milchindustrie eG (Palting, Germany). The microbial strains used in this study were selected in preliminary work (Table 1; [26]). The media for microbiological analyses were obtained from Scharlab S.L. (Sentmenat, Spain), and all chemicals used in the study were of analytical grade.

### 2.2. General Methods

#### 2.2.1. Strain Management

The lactic acid bacteria (LAB) were cultivated or enumerated in de Man-Rogosa-Sharpe (MRS) broth or agar as required; incubation was performed at 30 °C for 48 h under anaerobic conditions. The yeasts were cultivated or enumerated in yeast peptone dextrose (YPD) broth or agar medium; aerobic incubation was done at 26 °C for 48–72 h.

Long-term highly concentrated stock cultures were prepared in the respective media with the addition of 20 mL/100 mL glycerol and stored at −80 °C. Suspensions of fresh cells of each bacterial strain at approximately 10^9^ CFU/mL were obtained by cultivation in MRS broth under static conditions, while suspensions of fresh yeast cells at approximately 10^8^ CFU/mL were obtained by cultivation in YPD under agitation at 120 rpm. After centrifugation at 8000× *g* for 20 min (Rotina 380 R, Andreas Hettich GmbH & Co. KG, Tuttlingen, Germany), washing and resuspension in tryptone salt broth or peptone water for bacteria and yeasts, respectively, cells were enumerated by the plate count technique in the respective agar media. Fresh cultures were stored at 4 °C for a maximum of 1 wk before use.

#### 2.2.2. Fermentation Experiments

Following the protocol of Raak et al. [27], a blend of 22.5 g/100 g PC, suspended in whey powder reconstituted in demineralized water to 6 g/100 g dry matter, served as a fermentation substrate. The mixture was stirred for 10 min using a CNUM5ST planetary mixer (Robert Bosch GmbH, Gerlingen, Germany), followed by a thermal treatment at 112 °C for 10 min in 1 L screw-capped jars and cooling to room temperature in a cold room.

Under aseptic conditions in a laminar flow cabinet, co-cultures of lactic acid bacteria and yeasts (for details, see the respective experiments) were mixed into the blends to ensure an inoculation strength of 10^6^ CFU/g and 10^5^ CFU/g for LAB and yeasts, respectively. Fermentation was carried out under static conditions at 26 °C for 24 h or, in the second part of the study, until pH 4.80 was reached, and stopped by cooling in ice water. After taking a sample aliquot for microbial safety assessment, the fermented blends were shipped to the partners performing the sensory experiments by overnight courier and stored there at 5 °C until analysis.

#### 2.2.3. Assessment of Microbial Safety

All samples intended for sensory analysis were subjected to microbiological examination. The following microbial groups were enumerated using the colony count technique after appropriate decimal dilution in tryptone salt broth or ¼ strength Ringer’s solution: LAB by using MRS agar, anaerobic incubation at 30 °C for 48 h [31]; presumptive *Bacillus cereus* in polymyxin pyruvate egg yolk mannitol bromothymol agar, aerobic incubation at 30 °C for 48 h [32]; presumptive *Staphylococcus aureus* in Baird Parker agar plus supplements, aerobic incubation at 37 °C for 48 h [33]; presumptive *Escherichia coli* in tryptone bile glucuronic agar, aerobic incubation at 44 °C for 24 h [34]; yeasts and molds in chloramphenicol glucose agar, aerobic incubation at 25 °C for 72 h [35].

The potential presence of *Listeria monocytogenes* and *Salmonella enterica* in a 25 g sample was investigated by enrichment and subsequent detection in selective media, following the respective protocols [36,37].

#### 2.2.4. Chemical and Physical Analysis of Fermented Blends

pH was measured using a Jenway 3510 Standard Digital pH meter (Cole-Parmer Instrument Company LLC, Vernon Hills, IL, USA). Sugar, ethanol and organic acid content of the samples was determined using an Alliance 2695 HPLC pump system (Waters Corporation, Milford, MA, USA) equipped with a model 2414 differential refractive index detector. Prior to injection, samples were centrifuged at 19,600× *g* for 30 min at 4 °C in a Sorvall^®^ RC-5B centrifuge (Du Pont Inc., Wilmington, DE, USA).

For analyzing glucose, galactose, lactose, raffinose and sucrose, solutions containing 3.75 mL/100 mL sample supernatant and 15 mL/100 mL Biggs reagent [38] were made up to volume in graduated flasks with MilliQ water, allowed to decant for 45 min at 4 °C and then filtered through 0.22 μm membrane filters (Merck KGaA, Darmstadt, Germany). Sugar separation was performed using two Aminex HPX-87P columns in series (300 mm i.d. × 7.8 mm, Bio-Rad Laboratories Srl., Segrate, Italy), maintained at 75 °C, flow rate 0.6 mL/min, eluent MilliQ water, injection volume 5 μL.

For ethanol and organic acids analysis, solutions containing 5 mL/100 mL sample supernatant were prepared in graduated flasks made up to volume with 5 mmol/L sulfuric acid and filtered through a 0.22 μm membrane filter (Merck KGaA, Darmstadt, Germany). Separation of ethanol and organic acids was performed with the same columns, maintained at 50 °C, flow rate 0.6 mL/min, eluent 5 mmol/L H_2_SO_4_, injection volume 2 μL.

Aqueous solutions of acetic and lactic acid, ethanol, glucose, galactose, lactose, raffinose and sucrose served as external standards for the quantification of the investigated compounds. All analyses were performed in triplicate.

Viscosity measurements were carried out using a Physica MCR302 rheometer (Anton Paar GmbH, Graz, Austria) equipped with a serrated plate system (diameter, 40 mm; gap height, 1 mm) adjusted to 25 °C using Peltier temperature equilibration. Flow curves were recorded in duplicate in a shear rate window of 10^−2^/s to 10^2^/s.

## 3. Experiment 1: Initial Sensory Screening

### 3.1. Experimental Details

#### 3.1.1. Fermentation of Sunflower Press Cake/Whey Blends

Fermentation of the blends, made in two consecutive batches for initial free choice profiling (batch #1) and, after evaluation of the results, for duo-trio tests and quantitative descriptive analysis (batch #2), was carried out using six pairs of possible LAB and yeast combinations, namely B12|L2, B12|L12, B13|L2, B13|L12, B15|L2 and B15|L12 (see Table 1). The pH of the individual unfermented samples, used as control, ranged from 6.19 to 6.27.

#### 3.1.2. Sample Assessment by Free Choice Profiling

The six fermented samples and the control were initially subjected to free choice profiling (FCP), with details outlined by Jaros et al. [39]. In brief, 16 panelists who had passed the standardized training exercises specified in ISO 8586 [40] contributed to the experiment. The subjects were familiar with the FCP procedure and received information on the samples under study. All tests were performed in individual booths of a sensory laboratory, illuminated by red light. The panelists received approx. one tablespoon of each sample in 100 mL glasses, encoded with random three-digit numbers, and closed with a plastic lid. For administration, stainless steel teaspoons were used. Water and unleavened matzo-type bread were provided for neutralization.

In FCP session #1, the subjects were asked to use their individual, free choice vocabulary for describing the sensory key features of the samples from the fermentation batch #1. They were instructed to check appearance, smell and texture before tasting, as well as texture and flavor during tasting and the respective aftertaste impressions. The experimenter collected the individual descriptors (in German) and prepared A4 sheets with 14 unstructured scales (lines with 15 cm length, anchored by ‘not detectable’ and ‘very highly detectable’; [41]). In FCP session #2, performed on the consecutive day, each subject received a score sheet with their individual descriptors and then performed comparative ratings by placing marks on the scales and adding the respective sample code.

The FCP data, i.e., the readings taken from the unstructured lines, were analyzed by generalized procrustes analysis (GPA) using Senstools V.3.0.11 (OP & P Product Research BV, Utrecht, The Netherlands). Each of the 16 individual matrices had seven rows, whereas the respective number of columns corresponds to the number of descriptors elaborated by each assessor. The consensus matrix derived from individual data serves as a basis for principal component analysis, aiming at reducing matrix dimensionality with a minimum loss of variation [42].

#### 3.1.3. Distinguishability of Fermented Blends

In a subsequent experiment using the samples from fermentation batch #2, we focused on the distinguishability of fermented blends with similar acidity. To do so, specimens of B12|L2 (pH 4.54) and B12|L12 (pH 4.59), as well as less acidic systems B13|L2 (pH 5.20) and B13|L12 (pH 5.29) (see Table 1 and Table 2), were used for designing duo-trio tests. Thirteen of the trained subjects contributed to this part of the study, and each sample set was evaluated twice, again under red light, thus giving a total of 26 examinations [43]. Out of two candidate samples, the panelists were asked to identify the one different from the reference blend and to specify the descriptor most important for finding their choice.

#### 3.1.4. Quantitative Descriptive Analysis

Using descriptors identified in the FCP experiment and those relevant for sample differentiation, a quantitative descriptive profiling experiment (QDA) was performed. Fifteen panelists, all of them part of the FCP experiment, contributed here. The fermented blends were taken from batch #2, except for starter pair B15|L12 (see Table 1), for that, a frozen sample from batch #1 was used. The questionnaire comprised four unstructured scales 15 cm in length, namely ‘acidic’, ‘sunflower seed flavor’, ‘fermented, yeasty’ and ‘bitter’, that were labeled with anchors ‘not detectable’ and ‘very largely detectable’. The subjects received the samples, again encoded with three-digit numbers, as a complete set and were allowed to go back to already tested samples for comparison reasons. After taking the readings from the unstructured scales, the responses of each panelist per scale were normalized to zero mean and unit standard deviation [44].

### 3.2. Results

#### 3.2.1. Fermentation Characteristics

The fermented blends had a pH ranging from 4.45–5.20, with microbial associations containing *L. citreum* B13 mostly showing a pH > 5.0 due to the heterofermentative metabolism of this species (Table 2). Furthermore, the pH of the fermented blends was lower when *K. lactis* (L2) was inoculated, as this yeast is able to consume lactose and produce some lactic acid, which is not the case for *S. cerevisiae* (L12). On average, the LAB concentration exhibited an increase in three log units during fermentation, while that of the yeasts rose by two log units.

To ascertain the consumption suitability of the fermented blends, the microbiological criteria adopted in European legislative frameworks were used. Molds, *Enterobacteriaceae*, presumptive *E. coli* and *S. aureus* were never detected (<10^2^ CFU/g), and *L. monocytogenes* and *S. enterica* were always absent in 25 g of the sample. With the exception of one sample, which was consequently eliminated from investigation, the count of the presumptive *B. cereus* was below the detection limit (<10^2^ CFU/g). However, colonies of aerobic spore-forming bacteria were frequently observed at approximately 10^3^ CFU/g, which is consistent with previous reports for the same matrices [26].

#### 3.2.2. Sensory Discrimination of the Fermented Blends

**Figure 1:**
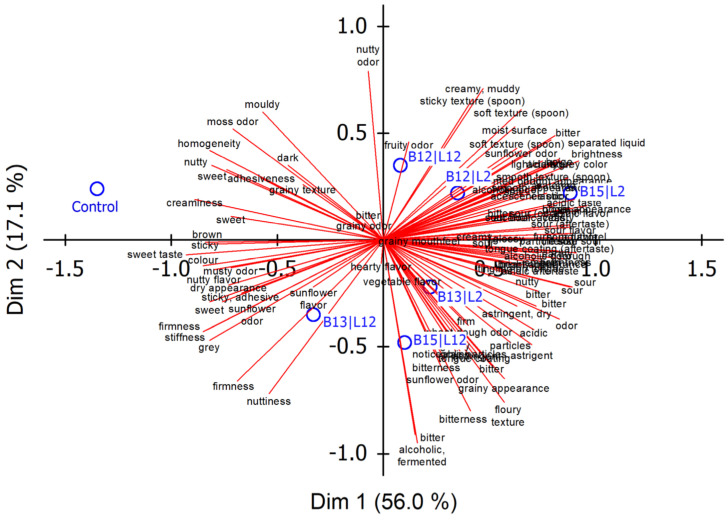
GPA group average plot for the individual descriptors obtained by free choice profiling. The unfermented reference and the fermented samples are also displayed in the consensus space. Identification of fermentation microorganisms: B12, *L. lactis*; B13, *L. citreum*; B15, *P. pentosaceus*; L2, *K. marxianus*; L12, *S. cerevisiae*. Control, unfermented blend.

In the first part of the FCP procedure, between 3 and 12 descriptors (median: 7) were generated by the assessors. The number of descriptors is lower than that observed for popular commodities such as drinks and beverages [41,42,45] but higher than that obtained for, e.g., chocolate [46]. Attributes most frequently chosen by the respondents during the first session were mostly related to texture, for example, ‘grainy’, ‘sticky’, ‘adhesive’ and ‘firm’, and flavor (‘sweet’, ‘sour’, ‘nutty’, ‘sunflower seed flavor’, ‘bitter’, ‘fermented’). The biplot, with the first two dimensions accounting for 73.1% of the total variance, is displayed in Figure 1. On the first principal axis, it is mainly the attributes ‘sweet’ versus ‘sour’ as well as ‘creaminess’ and ‘homogeneity’ versus ‘grainy’ and ‘phase separation’ that were found along the negative and positive coordinates, respectively. The attributes ‘bitter’ and ‘fermented’ were mainly present in the 4th quadrant of the biplot. The individual fermented samples are well distinguished, but it is also evident that the unfermented spread largely affected the spatial distribution of the samples. It is the blends fermented by *L. citreum* (B13) and, especially, those fermented by *P. pentosaceus* (B15) that, according to their position on the consensus plot, appeared as being influenced by the type of yeast used in fermentation.

As the GPA plot clearly showed that the unfermented sample was a main factor for distinguishing blends, the analysis was repeated after eliminating the data on the unfermented sample. We surely know that this is critical, as the unfermented sample also contributed to establishing descriptors, but, on the other hand, we were interested to know whether the relative positions of the blends fermented by the different consortia are comparable. It is evident from the respective GPA plot (Figure S1, descriptors not translated) that omitting the most deviating sample affects the consensus space, but the main outcome can be regarded as almost similar. The blends fermented using *L. lactis* (B12) appeared as only marginally affected by the yeast, whereas their influence on the sensory characteristics was more pronounced when *L. citreum* (B13) or *P. pentosaceus* (B15) were used as starter bacteria.

In the duo-trio discrimination test, the subjects were not able to distinguish the blends from batch #1 that were fermented using *L. lactis* and either of the yeasts. On the other hand, 19 out of 26 comparisons of the blends fermented using *L. citreum* and either *S. cerevisiae* (B13|L2) or *K. marxianus* (B13|L12) were correctly identified, indicating a difference on the *p* < 0.05 significance level [47]. The most occurring descriptors the subjects used to distinguish between the samples were ‘acidic’, ‘dry’, ‘bitter’, ‘sunflower seed flavor’, ‘nutty’, ‘fermented, yeasty’ and ‘astringent’.

**Figure 2:**
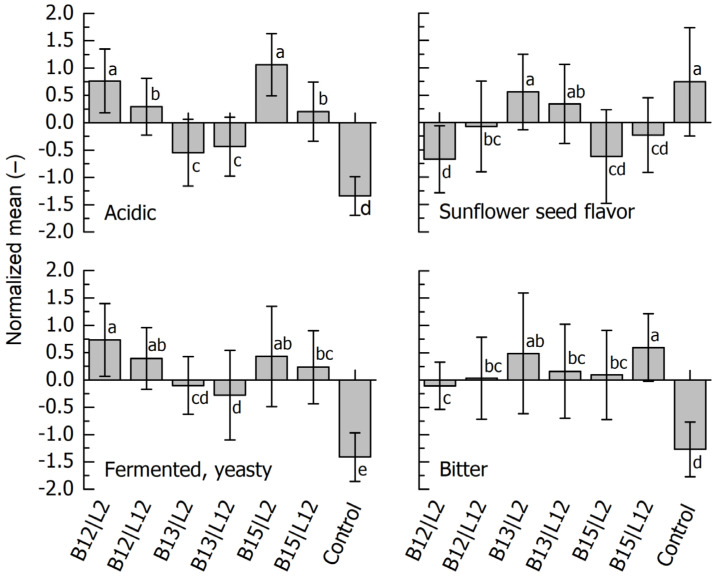
Quantitative descriptive analysis of fermented blends of sunflower press cake and whey. Identification of fermentation microorganisms: B12, *L. lactis*; B13, *L. citreum*; B15, *P. pentosaceus*; L2, *K. marxianus*; L12, *S. cerevisiae*. Control, unfermented blend. Mean values with different letters are significantly different (*p* < 0.05).

After discussing the results with the entire panel, the descriptors ‘acidic’, ‘sunflower seed flavor’, ‘fermented, yeasty’ and ‘bitter’ were chosen for the subsequent QDA experiment. The respective results summarized in Figure 2 show that there was a significant difference between the samples. As regards acidity, all blends with pH < 4.80 were rated above average. Blends B13|L2 and B13|L12 (pH 5.20 and 5.29, respectively; see Table 1) were scored significantly lower, as was the unfermented blend. ‘Sunflower seed flavor’ was more prominent in the less acidic samples. As regards the descriptor ‘fermented, yeasty’, we observed a tendency towards higher ratings for the blends where *K. marxianus* (L2) was present in the co-culture. Apart from the ratings of the unfermented reference blend, the bitterness results were less distinct. It also appears that it is mainly the difference in bitterness between B13|L2 and B13|L12 that was responsible for distinguishing the samples in the duo-trio test.

Regression analysis showed a significant correlation between sensory acidity and pH (*r* = –0.95, *p* < 0.05). The correlation matrix within sensory descriptors showed no relation between sensory bitterness and any other descriptor. The higher the acidity, the higher the rating for ‘fermented, yeasty’ (*r* = 0.92, *p* < 0.05) and the lower the rating for ‘sunflower seed flavor’ (*r* = –0.97, *p* < 0.05). Finally, the correlation coefficient between ‘sunflower seed flavor’ and ‘fermented, yeasty’ was *r* = –0.86 (*p* < 0.05).

#### 3.2.3. Fermented Blend Viscosity

The flow curves of the seven samples subjected to QDA were fitted using the Herschel-Bulkley model. The blends, including the unfermented one, were significantly shear thinning and showed a flow exponent of 0.12 < *n* < 0.14. The *k*-value, which corresponds to the apparent viscosity at a shear rate of 1/s, ranged between 0.56 × 10³ Pa.s and 0.92 × 10³ Pa.s. Viscosity of the fermented blends is comparable to that of fresh cheese with a protein and dry matter content of approx. 120 g/kg and 190 g/kg, respectively [48].

### 3.3. Conclusions and Implications for Subsequent Testing

The following conclusions were drawn from the results of the first set of experiments and used as a basis for designing the further tests:

- Time-based fermentation turned out to be unfeasible because of the different acidification activity of the involved LAB, and for the subsequent experiments, it was decided to ferment until pH 4.80.
- *L. citreum* (B13) acidified the slowest, to a pH significantly higher than for the other LAB. This might be a risk concerning product hygiene and safety, so this strain was not considered further.
- The samples fermented with mixtures of LAB and *K. marxianus* (L2) were more intense with respect to the ‘fermented, yeasty’ characteristic and described as more unpleasant by the subjects. Therefore, L2 was no longer considered.
- Furthermore, we decided to include a preliminary washing step for reducing bitterness induced by phenolic compounds present in the native press cake.
- The blank, unfermented sample appeared as not appropriate for being compared with fermented blends. After performing some minor experiments, it was decided to include a blend acidified to pH 4.80 by lactic acid, the main metabolite of the LAB, as a more appropriate control sample.

## 4. Experiment 2: Selecting Blends for the Final Sensory Evaluation

### 4.1. Experimental Details

#### 4.1.1. Preparation and Fermentation of Sunflower Press Cake/Whey Blends

To reduce bitterness caused by the press cakes’ phenolic compounds [30], the blends were prepared as reported by Mendo et al. [49]. A suspension of 10 g/100 g PC in demineralized water was stirred at 480 rpm for 10 min, followed by centrifugation at 8000× *g* for 30 min. The resulting pellet comprised 60% of the final blend, maintaining a total solids content of 22.5 g/100 g. The dry matter of the PC pellet was determined by the oven method [50]. The sweet whey powder was reconstituted at a concentration of 6 g/100 g in demineralized water, forming 40% of the final mixture, and this part brought a solids content of 2.4 g/100 g. The mixture was then kneaded for 10 min in the planetary mixer, sterilized at 121 °C for 15 min and cooled.

Fermentation was carried out using mixtures of *L. lactis* and *S. cerevisiae* (B12|L12, duplicate fermentation) and a mixture of *P. pentosaceus* and *S. cerevisiae* (B15|L12). In addition, another blend was fermented solely by using *L. lactis*. All fermentations, performed under static conditions at 26 °C, were stopped at pH 4.80. Blends of either washed or unwashed PC in whey, acidified with 80% L-lactic acid (Merck KGaA, Darmstadt, Germany) to pH 4.80, served as controls.

#### 4.1.2. Sensory Evaluation

In the first step of this part of the study, we used duo-trio tests to obtain information on whether fermentation is reproducible concerning sensory distinguishability and whether the washing step affects the sensory characteristics of PC/whey blends acidified by lactic acid. Fifteen of the trained subjects contributed to the experiment, and each panelist was asked to evaluate each of the two sample sets twice.

Subsequently, all 16 subjects from Experiment #1 contributed to QDA. Samples tested were the blends fermented by combinations of B12|L12 and B15|L12 (see Table 1), the blend fermented by solely using B12, and the not-inoculated blend of washed PC in whey acidified by lactic acid.

### 4.2. Results

**Figure 3:**
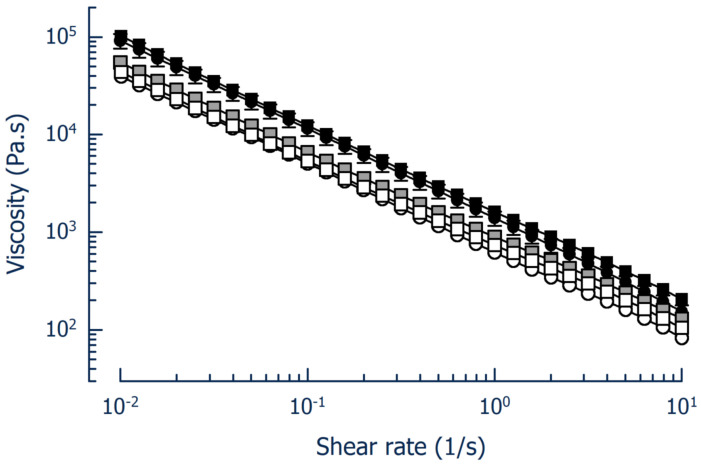
Flow curves of press cake (PC)/whey blends acidified either by fermentation or by direct acidification using lactic acid. Open circles, chemically acidified blend with unwashed PC; closed circles, chemically acidified blend with washed PC; squares, blends with washed PC fermented by *L. lactis* and *S. cerevisiae* (B12|L12, white), *P. pentosaceus* and *S. cerevisiae* (B15|L12, grey) or *L. lactis* (B12, black).

The LAB counts in the fermented blends reached approximately 2.0 × 10^9^ CFU/g, while the yeast counts were approximately 1.3 × 10^7^ CFU/g. As concerns either the washed or the unwashed and not inoculated controls, the results of the hygienic markers and pathogen determination were acceptable, as were the results of the fermented blends. The pH of all samples, either fermented or unfermented, measured immediately before sensory assessments, ranged from 4.81 to 4.93. Whereas the slope in the log viscosity versus log shear rate plot was comparable, the Herschel-Bulkley *k*-value of the samples was higher than in the first experiment (Figure 3). In detail, *k* was 0.62 × 10^3^ Pa.s and 1.39 × 10^3^ Pa.s for the chemically acidified blends made from unwashed or washed PC, respectively. For the fermented systems, viscosity at a shear rate of 1/s was 0.74 × 10^3^ Pa.s (fermentation with B12|L12; see Table 1), 0.89 × 10^3^ Pa.s (fermentation with B15|L12) and 1.61 × 10^3^ Pa.s (fermentation with B12). The higher viscosity can be attributed to the design of the washing procedure of the PC, which may have affected microstructural properties of the blends.

The results of the sensory discrimination tests were unequivocal. When comparing the two replicate fermentations with the B12|L12 consortium (see Table 1), 17 out of 30 single decisions in the one direction correspond to an insignificant *p* = 0.29. On the other hand, 25 out of 30 decisions (*p* < 0.001) were similar when the chemically acidified blends with either washed or unwashed PC were compared. The main comment provided by the subjects was on the different bitterness of the samples, with a higher intensity in the unwashed blend.

**Figure 4:**
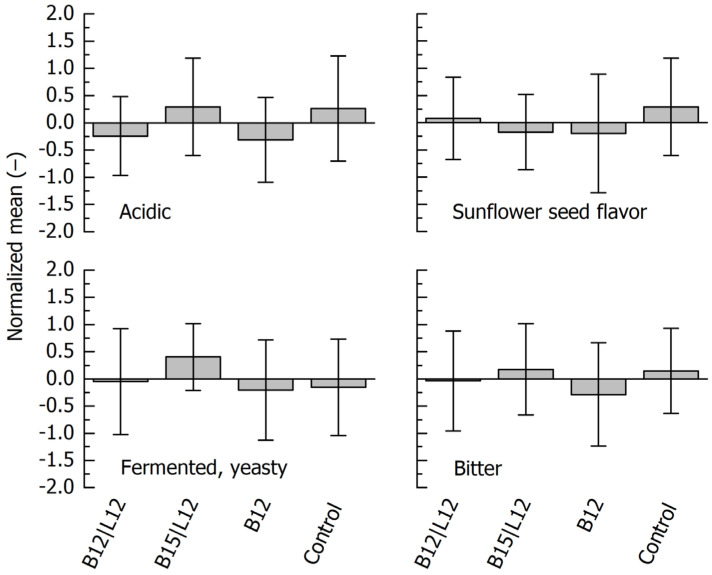
Quantitative descriptive analysis of fermented blends of sunflower press cake (PC) and whey. Identification of fermentation microorganisms: B12, *L. lactis*; B15, *P. pentosaceus*; L12, *S. cerevisiae*. Control, blend with washed PC acidified with lactic acid.

The QDA results are summarized in Figure 4. These appear as almost homogeneous, and no statistical differences (*p* < 0.05) between the samples were observed. It is, however, evident that the main deviation concerning ‘Sunflower seed flavor’ can be attributed to the unfermented specimen, and the main deviation concerning the descriptor ‘fermented, yeasty’ is caused by the sample fermented with a consortium of *P. pentosaceus* and *S. cerevisiae*.

### 4.3. Conclusions and Implications for Subsequent Testing

The following conclusions were drawn from the results of this section and used for designing the final consumer tests:

- Because of the explicit result of the initial discrimination test, including the verbal descriptions of the significant difference, it was decided to use blends of washed sunflower press cake and whey for the final consumer study.
- To allow including a sample for evaluating scoring consistency without introducing too much stress to the panel, we decided to eliminate also the B15|L12 consortium (*P. pentosaceus* + *S. cerevisiae*), mainly because of the ‘fermented, yeasty’ impression observed in QDA. In addition, its incubation time of approx. 70 h to reach pH 4.80 was considered as being too long.
- Based on a small questionnaire given to the subjects in the QDA test (details not given here), it was decided to include ‘Spreadability’ as the most important characteristic when using spread as a descriptor for a subsequent Check-all-that-apply test.

## 5. Experiment 3: Consumer Tests

### 5.1. Experimental Details

#### 5.1.1. Preparation of Blends

Blends of initially washed sunflower PC and whey (for composition, please refer to Section 4.1.1.) were fermented using a combination of *L. lactis* and *S. cerevisiae* (B12|L12) or by using only *L. lactis* (B12). In addition, the unfermented blend containing washed PC was acidified by lactic acid to pH 4.8.

#### 5.1.2. Sensory Evaluation

The last set of samples was evaluated using an acceptability test, a Check-all-that-apply (CATA) setup and, finally, a Just-about-right (JAR) design to obtain more detailed insight into the spreadability of the fermented blends. Seventy-eight panelists (ages 25 to 65 years, 55 female and 23 male) recruited at the entire Institute of Agrochemistry and Food Technology (IATA-CSIC) were invited to evaluate the respective sensory characteristics. All tests were conducted in a standardized sensory evaluation room with 10 separate booths, and the data were collected using the Compusense Cloud software (Compusense Inc., Guelph, ON, Canada). Samples were prepared the day before and refrigerated at 4 °C. After a waiting period of 2 h for equilibrating the sample’s temperature to ambient conditions, four samples [two control samples, *L. lactis* (B12) and *L. lactis* + *S. cerevisiae* spreads (B12|L12)], served in 30 mL glasses of 35 mm in diameter and covered with a lid, and identified with a random computer-assigned code, were tested. The unfermented but acidified control sample was presented twice to assess the consumers’ scoring consistency. To avoid any duplicate sample bias, the order the samples were presented to the consumers was also randomized. For assessing spreadability, a plastic knife was provided for spreading the samples onto a cracker.

A nine-point hedonic scale from 1 (‘dislike extremely’) to 9 (‘like extremely’) was used to measure the liking of color, smell, spreadability, texture, taste and global acceptability. Before each test, the participants were instructed to wait at least a 30 s interval and to rinse their mouth with water before evaluating the next sample to minimize interference between samples.

After that, consumers were asked to respond to a CATA questionnaire comprising 16 sensory descriptors, referring to appearance, texture, smell and taste. These were: ‘dark’, ‘wet’, ‘homogeneous’, ‘lumpy’, ‘spreadable’, ‘thick’, ‘off flavor’, ‘milky smell’, ’intense smell’, ‘legume smell’, ‘yeasty smell’, ‘acidic’, ‘bitter’, ‘yeasty taste’, ‘milky taste’, ‘astringent’. Three statements about the samples, such as ‘I presume it as healthy’, ‘I would buy it’ and ‘I would eat it as snack’, were also included. Each consumer was asked to check the terms that he/she considered appropriate to describe the spread samples.

Finally, sensory spreadability was measured using a JAR scale. Such a scale usually has five points to assess whether the level of a particular attribute is ‘Much too low’ (1), ‘Much too high’ (5) or ‘Just-about-right’ (3) [51]. The endpoint anchors represent levels of the attribute that deviate from a respondent’s theoretical ideal point in opposite directions, while the central point is the ideal [52].

Statistical analysis of all tests was carried out using XLSTAT 2019 1.1 (Addinsoft, Barcelona, Spain). The results of the acceptability test were analyzed by a one-way analysis of variance. Significant differences between means (*p* ≤ 0.05) were analyzed with Tukey’s multiple mean test. The non-parametric Cochran’s test analysis of variance was performed for each descriptor to evaluate whether the CATA question was able to detect differences in the consumer’s perception of the spreads. Overall variability in the frequencies of mention of significant attributes was analyzed by using a Correspondence Analysis (CA). For JAR analysis, an attribute was considered significant when the respondent percentage was higher than 20% [53].

### 5.2. Results

#### 5.2.1. Chemical Analyses

**Figure 5:**
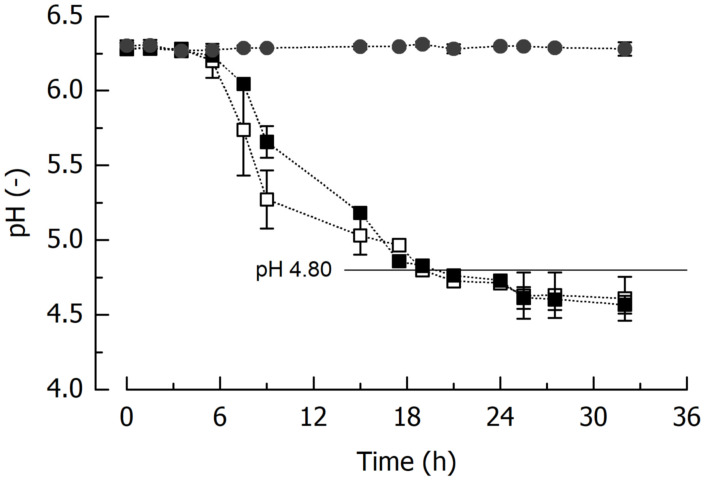
Acidification time course of blends of washed press cake and whey induced. Fermentation was done with *L. lactis* and *S. cerevisiae* (B12|L12, white squares) or *L. lactis* (B12, black squares). pH of the unfermented control (black circles) is also shown. Data is arithmetic mean ± standard deviation (*n* = 3).

The course of the fermentation process, depicted in terms of pH versus time, is shown in Figure 5. It is evident that both starter cultures performed almost identically independent of whether the yeast was present, that pH 4.8 was reached after fermentation for approx. 20 h, and that after this time point, acidification slowed down and the blend reached an equilibrium pH of approx. 4.6. The main fermentable sugar in the initial substrate was lactose, originating from the whey (Table 3). Approximately 25% of the lactose was metabolized during fermentation, giving a final concentration of approx. 1.55 g/100 g lactic acid. The same amount was needed to acidify the unfermented blend to pH 4.8. The content of acetic acid in the fermented blends was almost negligible, and ethanol content in the blend fermented using *L. lactis* and *S. cerevisiae* was 0.12 g/100 g, a concentration comparable to that in milk kefir [54]. Viable counts of LAB and yeasts were, where applicable, in the range that was observed in the first part of the study (see also Table 2).

#### 5.2.2. Hedonic Classification

**Figure 6:**
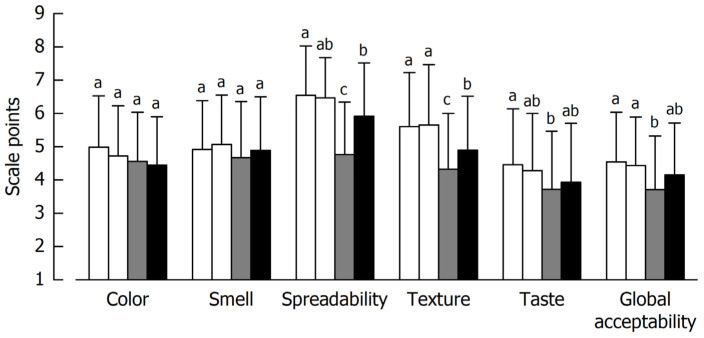
Sensory acceptability scores for the different spreads. White bars, controls #1 and #2 acidified with lactic acid; grey bars, blend fermented with *L. lactis* and *S. cerevisiae* (B12|L12); black bars, blend fermented with *L. lactis* (B12). Data are mean ± standard deviation (*n* = 78). Bars with different letters per characteristic differ significantly (*p* ≤ 0.05).

The results of the acceptability test are displayed in Figure 6. Both controls showed similar results, indicating sufficient and reproducible performance of the test panel. As concerns color and smell acceptability, no differences (*p* ≤ 0.05) were found among the samples. Overall, for the rest of the attributes, control spreads were more liked than the inoculated ones, with the sample co-fermented by *L. lactis* and *S. cerevisiae* (B12|L12) receiving lower scores than the blend fermented solely with *L. lactis*.

#### 5.2.3. Check-All-That-Apply Classification

**Figure 7:**
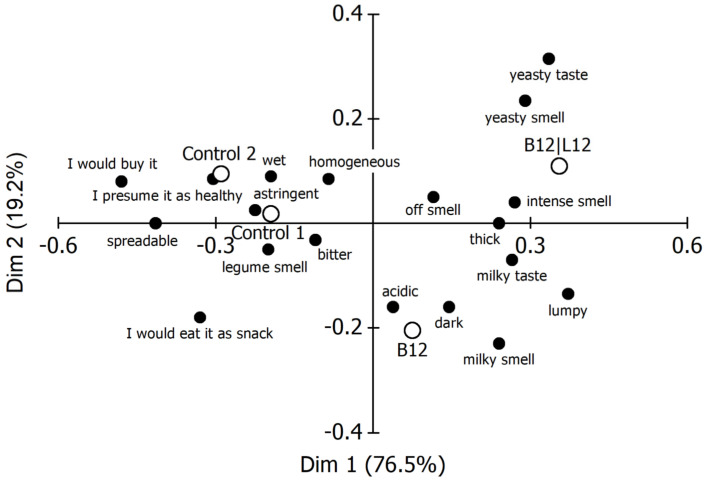
Correspondence analysis of the Check-all-that-apply responses in fermented spread evaluation. Controls #1 and #2, acidified with lactic acid. B12|L12, fermented with *L. lactis* and *S. cerevisiae*; B12, fermented with *L. lactis*.

The results from the CATA analysis are depicted in Figure 7. The analysis of the first two dimensions of the plot (95.7% of total variability) allowed distinguishing between the samples, as, according to their sensory attributes, these are located in three areas. The first dimension (responsible for 76.5% of total variability) separated both control samples from the fermented blends. Regarding texture and appearance, the panel members perceived the chemically acidified controls #1 and #2 as homogeneous, wet and spreadable, whereas, on the other hand, some described the fermented samples as thick and lumpy. Smell and taste were also scored differently. The panelists ascribed a legume-like smell and a bitter and astringent taste to the unfermented control blends and a yeasty and milky smell and taste as well as an acidic taste to the fermented samples. Finally, the consumers would buy and would eat the control samples as a snack and presume them to be healthy.

#### 5.2.4. Just-About-Right Scoring of Spreadability

The panelist’s comments during QDA, as well as the fact that the spreadability attribute was mentioned during CATA analysis for the control but not for the inoculated samples, and that it is a critical attribute for spreads, served as motivation for performing the JAR test. This helped to identify the direction for getting closer to an ‘ideal’ product and so to increase the overall liking for the respective spreads.

**Figure 8:**
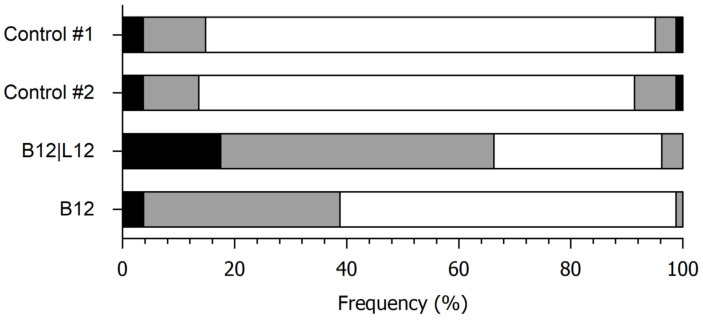
Just-about-right classification of the spreadability of the blends. Controls #1 and #2, acidified with lactic acid. B12|L12, fermented with *L. lactis* and *S. cerevisiae*; B12, fermented with *L. lactis*. Left and right black sections of the bars, spreadability assessed as much too low or much too high, respectively. Left and right grey sections of the bars, spreadability assessed as too low or too high, respectively. Central white section, spreadability assessed as just-about-right.

Figure 8 presents the fraction of consumers who identified the different levels of the attribute ‘spreadability’ as too low, just-about-right or too high. The averaged ratings for both controls were 2.88 ± 0.56 and 2.93 ± 0.60, both means not being significantly (*p* < 0.05) different from the scale midpoint. The fact that the a priori criterion of >70% in the just-about-right category is met indicates that there is no distinct need for optimization [54]. For the fermented blends under study, the mean ratings were 2.20 ± 0.76 (fermentation with *L. lactis* and *S. cerevisiae*) and 2.59 ± 0.59 (*L. lactis*). As levels with respondent percentages higher than 20% are significant to consumers, the inoculated samples were perceived as ‘too low’ spreadable, with the blend fermented by *L. lactis* + *S. cerevisiae*, the one in the sample set that showed the lowest viscosity, notably failing to reach the just-about-right level. There was, however, no indication for a bi-modal distribution, which would indicate that the respondents represent different segments of consumers [52]. The results of the JAR test also confirm the outcome of the hedonic tests (see Figure 6), although it was claimed that JAR scales show lower optimum levels [55,56]. It is also important to point out that CATA analysis showed that samples fermented with *L. lactis* or with the *L. lactis* and *S. cerevisiae* co-culture were associated with the attribute ‘thick’, indicating a high viscosity. Therefore, to improve their spreadability, these samples should be reformulated to achieve a more fluid texture.

## 6. Limitations

The authors are aware of a few limitations of this study, which should explicitly be mentioned. First of all, chemical acidification of a press cake/whey blend seems to be sufficient to reach acceptable products. It is, however, well known that fermentation is associated with bioprotective mechanisms against potential contaminants, for instance, the depletion of fermentable resources (mainly carbon and nitrogen) and the production of primary and secondary metabolites with antimicrobial activity (organic acids, alcohols, or bacteriocins). This is a confident and sustainable way to improve food safety and the nutritional quality of, especially, plant-based products. Concerning additional positive effects on the sensory and structural characteristics of press cake/whey blends through fermentation, it was a strategic choice inherent to the underlying research project, since the history of mankind is full of examples of enhancing food quality by the controlled use of microorganisms. This was not the case when applying co-fermentation with LAB and yeasts, but a careful investigation on fermentation with single strains of either mesophilic or thermophilic lactic acid bacteria might help to override this limitation.

It is also clear that the use of such a low number of (even trained) subjects, in line with repeated testing, can be seen as critical. In the context of this study, these duo-trio tests were performed to narrow down sample variability for subsequent experiments. Although a mixed panel comprising researchers, administrative and library staff and cleaning and maintenance services contributed to the respective tests, it is also the consumer tests that should be expanded to a broader basis before entering the market with such a product.

## 7. General Conclusions

The study demonstrates that it is possible to develop a fermented blend from sunflower press cake and whey for use as a basis for a savory spread. The results confirm that when using food matrices with a high contamination potential of spore-forming bacteria, product sanitation by sterilization is necessary. Furthermore, rapid acidification of the material by lactic acid bacteria provides additional protection against the development of contaminants and is therefore necessary for ensuring product safety. Contrary to popular opinion, the co-fermentation with LAB and yeasts does not always lead to the formation of pleasant aromatic compounds, presumably because of the relatively short fermentation time, which did not allow the formation of esters and higher alcohols. Washing of press cake prior to blending it with whey helped to significantly reduce the bitterness of the spreads. Results from all sensory experiments also demonstrated that such a blend fermented with appropriate microorganisms may serve as a basis for a savory spread, provided that further optimization concerning product texture and flavor is ensured.

## Figures and Tables

**Table 1:** List of microbial strains used in this work [26].

Code	Species	Origin
B12	*Lactococcus lactis*	Commercial milk kefir
B13	*Leuconostoc citreum*	Homemade sugary kefir
B15	*Pediococcus pentosaceus*	Blend of sunflower press cake and reconstituted whey
L2	*Kluyveromyces lactis*	Household milk kefir
L12	*Saccharomyces cerevisiae*	Homemade kvass

**Table 2:** Microbiology of samples co-inoculated with lactic acid bacteria (LAB) and yeasts.

Strain Association	B12|L2		B12|L12		B13|L2		B13|L12		B15|L2		B15|L12	
Batch	#1	#2	#1	#2	#1	#2	#1	#2	#1	#2	#1	#2
pH after fermentation	4.57	4.54	4.66	4.59	4.81	5.20	5.23	5.29	4.45	4.47	4.72	5.02
LAB (log CFU/g) at 0 h	6.60	5.91	6.66	6.00	6.74	6.10	6.73	6.10	6.06	6.06	6.16	6.09
LAB (log CFU/g) after fermentation	9.31	9.67	9.38	9.44	9.50	9.63	9.50	9.50	9.35	9.56	9.37	9.08
Yeasts (log CFU/g) at 0 h	4.96	5.06	5.34	5.08	5.06	5.14	5.12	5.09	5.00	5.06	5.16	5.19
Yeasts (log CFU/g) after fermentation	6.65	7.15	7.39	7.10	6.24	6.48	6.89	6.89	7.27	7.30	7.49	7.00
*B. cereus* (log CFU/g) at 0 h	<2.00	<2.00	<2.00	<2.00	<2.00	<2.00	<2.00	<2.00	<2.00	<2.00	<2.00	<2.00
*B. cereus* (log CFU/g) after fermentation	<2.00	<2.00	<2.00	<2.00	<2.00	<2.00	<2.00	<2.00	<2.00	<2.00	<2.00	>2.00 *

Strain encoding: B12, *L. lactis*; B13, *L. citreum*; B15, *P. pentosaceus*; L2, *S. cerevisiae*; L12, *K. marxianus*. * Sample in which presumptive *B. cereus* was observed.

**Table 3:** Chemical and microbial characteristics of samples co-inoculated with *L. lactis* (B12) and *S. cerevisiae* (L12), solely fermented with B12 or not inoculated but chemically acidified.

Title 1	B12|L12	B12	Control (Chemical Acidification)
**Sugars (g/100 g)** Galactose Glucose Lactose Raffinose Sucrose			
	n.d.	n.d.	0.11 ± 0.02
	n.d. 3.36 ± 0.09 n.d. n.d.	n.d. 3.37 ± 0.01 0.10 ± 0.05 0.18 ± 0.03	0.06 ± 0.02 4.53 ± 0.01 0.07 ± 0.04 0.14 ± 0.06
**Organic acids and alcohol (g/100 g)** Acetic acid Lactic acid Ethanol	0.02 ± 0.00 1.54 ± 0.12	0.02 ± 0.00 1.52 ± 0.07	n.d. 1.55 ± 0.03
	0.12 ± 0.03	n.d.	n.d.
**Viable counts (log CFU/g)** Lactic acid bacteria Yeasts			
	9.26	8.61	<2
	7.06	<2	<2

n.d., not detected.

## Data Availability

The original contributions presented in the study are included in the article/Supplementary Material, further inquiries can be directed to the corresponding author.

## Supplementary Materials

The following supporting information can be downloaded at: https://www.mdpi.com/article/10.3390/foods14091489/s1, Figure S1: GPA group average plot for the individual descriptors (not translated) obtained by free choice profiling. In contrast to Figure 1, only the fermented samples were considered in data evaluation. The fermented samples are also displayed in the consensus space. Identification of fermentation microorganisms: B12, *L. lactis*; B13, *L. citreum*; B15, *P. pentosaceus*; L2, *K. marxianus*; L12, *S. cerevisiae*.
