# Supplementary material for: Sensory Properties of Fermented Blends of Sunflower Press Cake and Whey
Source: Foods. 2025 Apr 24;14(9):1489. doi: 10.3390/foods14091489 (PMC12071512; doi:10.3390/foods14091489)
Supplement: Supplementary file 1 [file foods-14-01489-s001.zip › foods-3555988-supplementary.pdf]

Supplementary Information

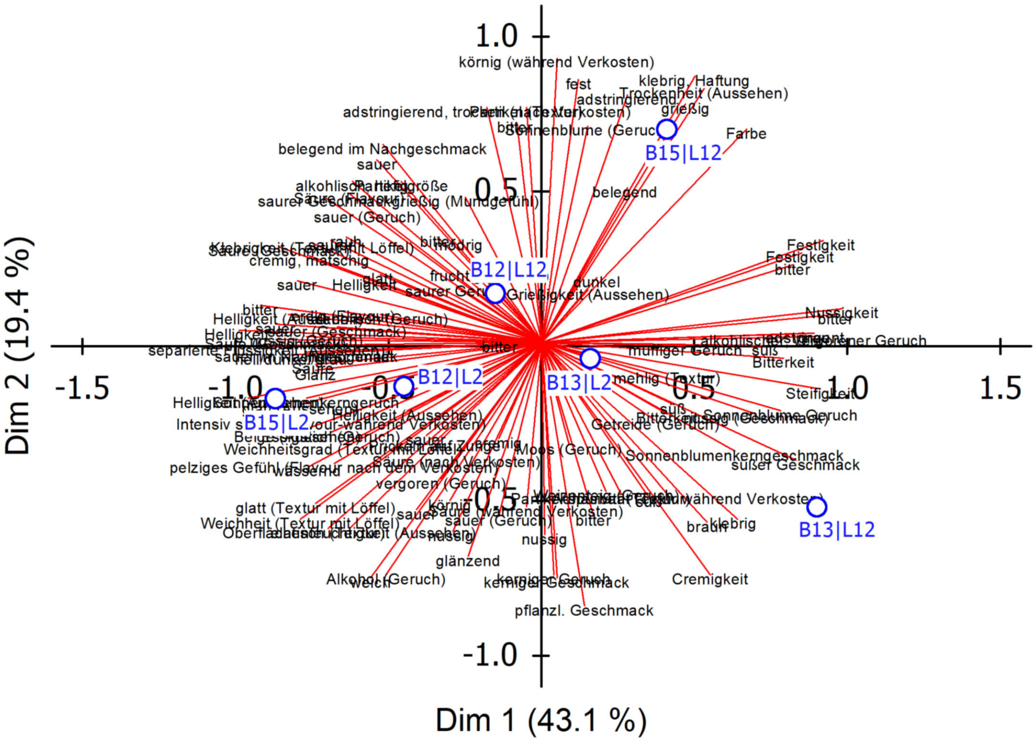

Figure S1: GPA group average plot for the individual descriptors (not translated) obtained by free choice profiling. In opposite to Figure 1, only the fermented samples were considered in data evaluation. The fermented samples are also displayed in the consensus space. Identification of fermentation microorganisms: B12, *L. lactis*; B13, *L. citreum*; B15, *P. pentosaceus*; L2, *K. marxianus*; L12, *S. cerevisiae*.
